# Loss of childcare and classroom teaching during the Covid-19-related lockdown in spring 2020: A longitudinal study on consequences on leisure behavior and schoolwork at home

**DOI:** 10.1371/journal.pone.0247949

**Published:** 2021-03-02

**Authors:** Tanja Poulain, Christof Meigen, Carolin Sobek, Peggy Ober, Ulrike Igel, Antje Körner, Wieland Kiess, Mandy Vogel

**Affiliations:** 1 LIFE Child, LIFE Leipzig Research Center for Civilization Diseases, Leipzig University, Leipzig, Sachsen, Germany; 2 Department of Women and Child Health, University Hospital for Children and Adolescents, and Center for Pediatric Research, Leipzig University, Leipzig, Sachsen, Germany; 3 Center for Research and Transfer (FTZ) at the Leipzig University of Applied Sciences (HTWK), Research Field Health and Social Affairs, Leipzig, Sachsen, Germany; Fordham University, UNITED STATES

## Abstract

**Aim:**

In spring 2020, the first Covid-19-related lockdown included the closing of kindergartens and schools. Home schooling, the lack of social contacts with peers and the care of the children at home posed an enormous challenge for many families.

**Methods:**

The present study investigated the leisure behavior of 285 one- to 10-year-old German children at two time points (t1 and t2) during the Covid-19-related lockdown in spring 2020. In the subsample of primary school children (n = 102), we also explored children’s attitudes towards schoolwork at home. Analyses focused on the change of behavior from t1 to t2, on differences in these changes depending on socio-economic status (SES), and on associations of behavior with SES, the number of children at home, and the frequency of receiving learning materials from school.

**Results:**

While the frequency of playing outside increased significantly from t1 to t2, the frequency of handicrafts, playing board games, indoor sports, and motivation to do schoolwork decreased. The observed changes between t1 and t2 did not differ depending on SES. However, a lower SES was associated with higher media use, less outdoor activity, and (though only marginally significant) a reduced time doing schoolwork and a reduced ability to concentrate on schoolwork at t1. In households with more children, children played outside more often, but were read to less frequently and (though only marginally significant) watched movies and series less frequently. Children receiving learning materials from school on a regular basis spent significantly more time doing schoolwork at home than children receiving materials only irregularly.

**Conclusions:**

A continuing loss of childcare in day-care facilities and schools entails the danger of declining education in the form of (inter)active indoor activities and schoolwork.

## Introduction

At the beginning of 2020, Covid-19 claimed numerous victims worldwide in a very short time. In March 2020, the WHO characterized the COVID-19 virus outbreak as a global pandemic. In order to prevent the virus from spreading further, various measures were taken, e.g., closures of public facilities and adoption of strict distance rules. Because of these measures, infection rates decreased. However, the lockdown was also a challenge, especially for families [1]. With the closure of childcare facilities, children lost their natural play and learning environment, schoolchildren were forced to do schoolwork from home and families had to cope with reduced mobility and personal space. In addition to possible health concerns and financial constraints, parents had to balance work, childcare, and school support.

Possible consequences of the pandemic, the resulting lockdown and the lack of daily routine in children and adolescents are numerous [2] and include fear and uncertainty [3], behavioral and mental health problems [4–7], an increase of abuse and neglect [8–11], and an increasingly unhealthy lifestyle [12–15]. Two studies conducted in regions with especially high infection rates (China [12] and Italy [13]) showed a significant increase in screen time and a decrease in physical activity in children during the lockdown in spring 2020. A study in children with congenital heart disease showed that the steps counted on weekdays in March/April 2020 (i.e., during the Covid-19-related lockdown) were significantly lower than on weekdays one year before [14]. Other problems associated with the closure of schools are difficulties in completing schoolwork and in adequately supporting children in doing their schoolwork. In a study conducted in the Czech Republic, parents of nearly 10.000 children and adolescents were asked to evaluate the efforts of homeschooling [16]. Nearly half of the participants stated that the amount of schoolwork is large; however, the large majority reported to cope well with the home learning situation.

As described above, several authors described possible consequences of school closure and social isolation on wellbeing and behavior. However, most of them did not present empirical data. The aim of the present study was to investigate the leisure behavior and the attitudes towards schoolwork in children living in Germany at two time points during the first Covid-19-related lockdown, once at the beginning and once at the end of school closures. In spring 2020, Germany–especially eastern Germany, the region where the present study was conducted–was less affected by the pandemic than other countries. Nevertheless, schools and childcare facilities were closed between mid-March and mid-May, and contact with people outside the home was banned. Schools were asked to provide the children with learning materials and schoolwork, preferably online. We expected to observe an increase in media use but a decrease in other leisure activities and school engagement from the beginning to the end of the lockdown. As other studies indicate that families from lower social strata are especially affected by the risks related to the pandemic and lockdown [5,17,18], we hypothesized that the expected changes during the lockdown were stronger in children from lower social strata. In general, we expected to observe an unhealthier lifestyle (higher media use, less physical activity) and more problems regarding schoolwork in children from families with a lower socioeconomic status (SES). Furthermore, we assessed if the number of children in the home is associated with leisure behavior and attitudes towards schoolwork. In families with more children, we expected a higher frequency of activities that may involve several children (e.g., board games), but a lower frequency of activities that require individual attention (e.g. being read to). With respect to school engagement, we also investigated associations with the frequency of receiving learning materials from school.

## Materials and methods

### Participants and design

Data was collected within the framework of the LIFE Child study, a longitudinal study examining healthy development from the prenatal stage to adulthood [19]. The LIFE Child study was approved by the Ethics Committee of the Medical Faculty of the Leipzig University (Reg. No. 264/10-ek) and was performed in accordance with the ethical standards as laid down in the 1964 Declaration of Helsinki and its later amendments. All parents provided informed written consent before the participation of their children in the LIFE Child study.

During the first Covid-19-related lockdown in spring 2020, all families who previously took part in the LIFE Child Study were invited to complete online surveys at two time points, once 1–2 weeks after the closure of schools and kindergartens (March/April 2020, t1) and one month later, when schools and kindergartens were still closed but a first relaxation of restrictions was underway (April/May 2020, t2). The present project focused on children aged one to 10 years whose parents had completed online questionnaires at both t1 and t2. At each time point, parents of a total of 1094 children were sent an e-mail with a personalized link to the survey. At t1 and t2, surveys of 641 and 571 children were sent back. Parents of 461 children had completed the online survey at both t1 and t2. All children visiting emergency childcare at t1 and/or t2 (n = 77) were excluded from the sample. Furthermore, only one child per family was selected at random, 99 siblings were excluded. The final sample comprised 285 children.

### Measures

The online surveys were developed for the LIFE Child study and contained questions on the burden for families and on the wellbeing and behavior of children. A selection of questions dealing with leisure behavior (handicrafts, board games, indoor sports, playing outside), media use (watching movies and series, playing video games, receiving reading/reading together, listening to music), the way home schooling was organized by schools (frequency and way of providing children with materials), and children’s attitudes towards schoolwork (concentration, fun, motivation) was analyzed in the present study (see S1 Table). All questions were answered by one parent. The SES of children was derived using data collected at the last study visit in the LIFE Child study (conducted between 2016 and 2020, with the majority of data (93%) from 2019 or 2020). The SES score combines information on parental education (school and professional), occupational status, and equalized household income. The score ranges between 3 and 21, with higher scores indicating higher SES, and can be categorized as low, middle, or high SES [20]. Given the low proportion of children from families with a low SES (< 1%), we combined low and middle SES into one category (low/middle SES). The number of children at home and the frequency of children receiving learning materials from school were assessed in the online survey.

### Statistical analysis

All analyses were performed using R [21]. Data was described in terms of numbers and percentages (for categorical variables) or means and ranges (for metric variables). Associations of leisure behavior, media use, and attitudes towards schoolwork with age and gender were assessed at t1 using linear or proportional odds logistic regression analyses. Linear or proportional odds logistic regression analyses were applied to assess differences between answers given at t1 and t2 and interactions between time point and SES (as continuous measure). Associations of leisure behavior and media use at t1 with SES and the number of children at home (as continuous measures) were also assessed using linear or proportional odds logistic regression analyses. For attitudes towards schoolwork, we also investigated associations with the frequency of receiving learning materials by the school (regularly (at least once/week) versus irregularly (less often than once/week)). All associations were adjusted for age and gender. Effects with a p < .05 were considered significant. Effects with a p < .1 were considered marginally significant.

## Results

### Data description

In total, the study sample comprised 285 children (152 boys, 133 girls). The mean age was 5.56 (range 1.44–10.69) at t1. Before the lockdown, the majority of children (n = 183, age 1.44–7.36) visited the nursery/kindergarten, and 102 visited primary school (age 6.61–10.70). During the lockdown, mothers were mainly responsible for childcare at home (in about 50% only the mother, in about 33% mother and father, see Table 1).

**Table 1:** Information on demographics, childcare situation, and type of school support at t1.

Measure		T1	T2
Age	Mean (range)	5.56 (1.44–10.69)	5.64 (1.53–10.78)
Gender	Male	152 (53%)	
	Female	133 (47%)	
SES	High	154 (54%)	
	Low/middle	131 (46%)	
Number of children at home	Mean (range)	1.98 (1–5)	
Childcare situation before lockdown	Nursery/kindergarten	183 (64%)	
	Primary school	102 (36%)	
Person responsible for childcare during lockdown	Mother	139 (49%)	148 (52%)
	Father	39 (14%)	34 (12%)
	Mother and father	95 (33%)	94 (33%)
	Grandparents	10 (4%)	6 (2%)
	Other	2 (0%)	3 (1%)
Frequency of receiving learning materials from school^a^	Regularly (at least 1x/week)	69 (68%)	81 (79%)
	Irregularly (less than 1/week)	33 (32%)	21 (21%)
Way in which schools provided children with learning materials^a^	Internet platform	29 (28%)	40 (39%)
	E-Mail	75 (74%)	84 (82%)
	Learning videos	16 (16%)	26 (25%)
	Other	34 (33%)	17 (17%)

^a^Assessed in the subsample of primary school children only (n = 102).

**Fig 1:**
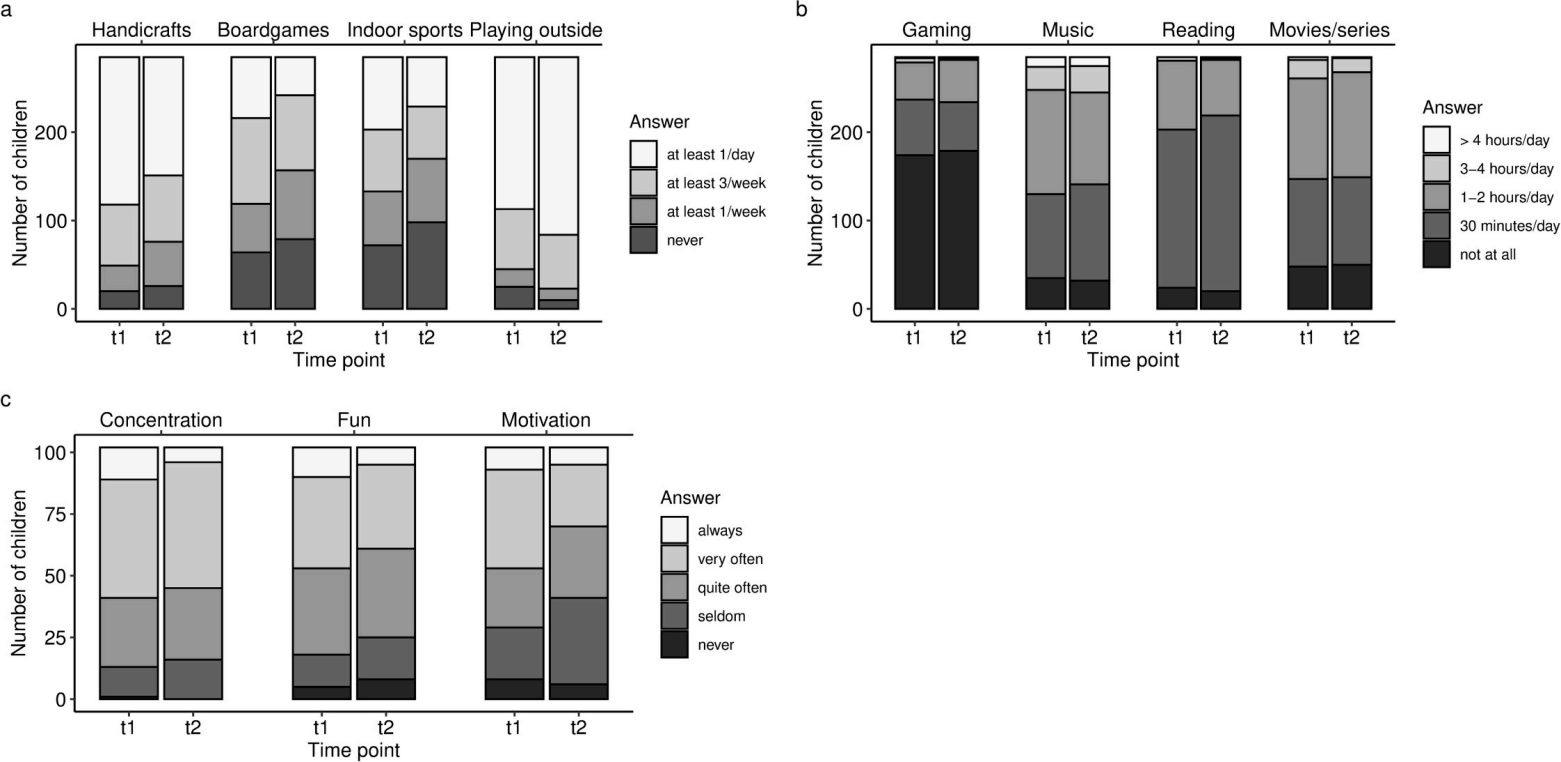
Leisure activities (a), daily media usage durations (b), and attitudes towards school (c) at t1 (beginning of lockdown in spring 2020) and t2 (1 month later). While the frequency of handicrafts, board games and indoor sports, and the motivation to do schoolwork decreased significantly from t1 to t2, the frequency of playing outside increased.

The distributions of SES, number of children at home, the frequency of receiving materials from school and the way in which schools provided children with materials are shown in Table 1. The distributions of leisure behavior, media use, and attitudes towards schoolwork are shown in Fig 1.

Regarding leisure activities, most parents reported that their children made handicrafts and played outside at least once per day. Board games and indoor sports were observed less frequently, with most parents stating that their children show these behaviors 1–3 times per week (Fig 1A). With respect to media use, listening to music and watching movies or series were the media activities engaged in most frequently (Fig 1B). At t1, 53 (49%) of all pre-school aged children watched movies or series for more than 30 minutes per day and, therefore, exceeded current recommendations [22]. Of the primary school children, 12 (12%) clearly exceeded the very loose recommendation to limit screen time to a maximum of 2 hours per day [22]. In contrast to the high frequencies of watching movies or series and listening to music, the daily duration of playing video games was rather low, with the majority of children playing no games at all.

In the subsample of primary school children, all children received materials for homeschooling provided by their schools. At t1, nearly 70% received learning materials on a regular basis. This percentage increased to over 80% at t2 (see Table 1). The way of providing pupils with learning materials also changed from t1 to t2 (see Table 1). Whereas the percentage of using internet platforms, sending materials via e-mail, and providing learning videos increased, “other” ways (e.g., having printed worksheets picked up at school) decreased. The average duration of schoolwork at home was 2.49 hours (range 0.5–5.00) at t1, and 2.67 (range 0.25–7.00) at t2. Concerning attitudes towards schoolwork at home, most parents stated that children were motivated, had fun, and were able to concentrate quite often or very often (Fig 1C).

A higher age of children was significantly related to a higher frequency of playing board games (OR = 1.27 (95% CI 1.17–1.39)), watching movies or series (OR = 1.36 (95% CI 1.25–1.49)), and playing video games (OR = 1.85 (95% CI 1.64–2.12)). In contrast, a higher age was associated with a lower frequency of handicrafts (OR = 0.83 (95% CI 0.76–0.91)), playing outside (OR = 0.83 (95% CI 0.76–0.91)), and receiving reading (OR = 0.74 (95% CI 0.67–0.82)). Compared to boys, girls were reported to engage more frequently in handicrafts (OR = 2.66 (95% CI 1.65–4.34)) and listening to music (OR = 1.89 (95% CI 1.23–2.92)), but less frequently in playing video games (OR = 0.54 (95% CI 0.31–0.93)). The other behaviors and attitudes were not significantly associated with age or gender.

### Differences between observations at t1 and t2

Table 2 illustrates the differences between the activities/attitudes at t1 and t2. As can be seen, the frequency of board games, handicrafts, and indoor sports decreased significantly from t1 to t2, while the frequency of playing outside increased (Fig 1A). At t1, the frequency of children playing outside daily was 60%, compared to 71% at t2. Furthermore, the motivation to do schoolwork decreased significantly (Fig 1C). At t1, 46% of parents stated that their children were able to motivate themselves often or always, compared to 34% at t2. The duration of media use and the duration of schoolwork, in contrast, did not change significantly between t1 (mean = 2.49) and t2 (mean = 2.67) (p = .333). The analyses revealed no significant interactions between time point and SES (all p > .05), indicating that the changes between t1 and t2 did not differ depending on SES.

**Table 2:** Differences between observations made at t1 and t2, and associations of observations made at t1 with SES, number of children at home, and the frequency of receiving learning materials from school.

	Differences t1—t2		Associations with					
			SES		Number of children at home		Frequency of receiving materials^a^	
	OR (95% CI)	*p*	OR (95% CI)	*P*	OR (95% CI)	*P*	OR (95% CI)	*p*
**Leisure activities**								
Board games	0.62 (0.46–0.84)	.002	0.96 (0.88–1.03)	.254	1.14 (0.87–1.51)	.344	-	-
Handicrafts	0.62 (0.45–0.85)	.003	1.03 (0.95–1.12)	.483	0.98 (0.72–1.33)	.878	-	-
Indoor sports	0.61 (0.46–0.83)	.001	0.96 (0.89–1.03)	.254	0.93 (0.71–1.21)	.584	-	-
Playing outside	1.65 (1.18–2.33)	.004	1.16 (1.07–1.26)	< .001	1.60 (1.17–2.22)	< .001	-	-
**Media activities**								
Gaming	0.95 (0.68–1.32)	.766	0.90 (0.82–0.99)	.026	0.92 (0.65–1.30)	.656	-	-
Music	0.94 (0.69–1.27)	.675	0.92 (0.85–0.99)	.033	0.94 (0.71–1.24)	.649	-	-
Reading	0.83 (0.59–1.18)	.302	0.99 (0.91–1.08)	.836	0.77 (0.63–0.95)	.046	-	-
Movies, series	0.93 (0.69–1.26)	.643	0.84 (0.77–0.91)	< .001	0.77 (0.58–1.01)	.063	-	-
**Schoolwork**^**a**^								
Concentration	0.75 (0.45–1.26)	.283	1.12 (0.99–1.26)	.083	0.79 (0.50–1.23)	.291	0.75 (0.35–1.60)	.451
Fun	0.68 (0.41–1.12)	.132	1.08 (0.95–1.21)	.228	0.82 (0.53–1.28)	.378	0.64 (0.30–1.36)	.247
Motivation	0.59 (0.34–0.97)	.039	1.12 (1.00–1.26)	.058	1.03 (0.65–1.64)	.901	0.60 (0.29–1.27)	.184

All associations were adjusted for age and gender.

^a^Assessed in the subsample of primary school children only (n = 102).

### Associations of observations made at t1 with SES, the number of children at home (and the frequency of receiving learning materials from school)

Children from families with a lower SES were reported to play outside less frequently and to spend more time using media (watching movies or series, playing video games, listening to music) than children from families with a higher SES (Table 2). In families with a low/middle SES, 51% of parents stated that their child played outside daily, compared to 68% in families with a high SES.

In the subsample of primary school children, a higher SES was associated with a higher ability to concentrate on schoolwork and a higher motivation to do schoolwork (Table 2). However, these associations were only marginally significant (p = .083 and .058, respectively). Similarly, SES showed a marginal significant positive association with the duration of daily schoolwork (b = 0.06 (95% CI -0.01–0.13), p = .096).

A higher number of children at home was significantly associated with a higher frequency of playing outside and with shorter durations of daily reading to children. In homes with one child, 54% of children were reported to play outside daily, compared to 70% in homes with three children. In addition, we observed an (only marginally significant) association between a higher number of children at home and a lower frequency of watching movies or series at home (p = .063). In contrast, the number of children at home was associated neither with attitudes towards schoolwork (Table 2) nor with the duration of daily schoolwork at home (b = 0.004 (95% CI -0.28–0.29), p = .216).

The frequency of receiving learning materials from school was significantly associated with the average daily duration of schoolwork at home (b = -.46 (95% CI -.91 to -.003), p = .046). In children receiving materials only irregularly, the duration of schoolwork was estimated to be 28 minutes shorter than in children receiving materials on a regular basis. However, the frequency of receiving learning materials was not associated with the attitudes towards schoolwork (Table 2).

## Discussion

The present study investigated the leisure behavior of one- to 10-year-old children at two time points during the period of Covid-19-related closures of childcare institutions in spring 2020. In the subsample of primary school children, we also explored the attitudes towards schoolwork.

### Leisure behavior during lockdown

On a positive note, the analyses showed that the majority of children played outside and did handicrafts every day. However, the data also showed a high frequency of watching movies or series, with half of the pre-school aged children exceeding current recommendations to limit the daily screen time to 30 minutes per day [22]. Overall, children’s media use was high, which is in line with recent studies showing long screen times in children and adolescents during the Covid-19-related lockdown [12,13,15]. The observations also confirm previous studies showing an increase of sedentary and a decrease of active behavior during school holidays, i.e., when external structures are missing [23,24].

As the closures of childcare facilities and homeschooling continued, the frequency of (indoor) leisure activities decreased while the frequency of playing outside increased. One possible reason for this finding is that better weather and a decreasing fear of the virus at the second compared to the first survey date led to a shift from indoor to outdoor activities. However, the frequency of media use, another indoor activity, did not change between the two survey points. This might indicate that the decrease in indoor activities is limited to more (inter)active behavior and activities demanding the involvement of parents. The longer a challenging family situation lasts, the more difficult it may be to find new ideas for exciting and at the same time pedagogically valuable indoor activities and to free up enough time and energy for parental engagement.

As expected, children from families with a lower SES were reported to play outside less frequently but to spend more time using electronic media than children from families with a higher SES. However, contrary to our hypotheses, changes observed between the beginning and the end of the lockdown did not differ depending on SES. Therefore, the associations between behavior and SES can be assumed to reflect a general trend, which has already been observed in other studies [25], rather than a phenomenon specific to (and increasing during) the Covid-19-related lockdown. This finding contradicts previous studies suggesting that families from lower social strata are especially affected by the risks related to an epidemic-related lockdown [5,17,18]. However, it has to be taken into account that the present study sample was not very large and included mainly children from higher social classes. It is often the families with the lowest SES (which are underrepresented here) that differ from families with medium or high SES.

In families with more children at home, children were reported to play more frequently outside but to receive less reading and to watch movies or series less frequently (the latter association was only marginally significant). These findings might be explained by a lack of time for the individual child in families with several children (of different ages). In a household with many children, there is less time for individual activities, e.g. reading of age-appropriate books, than in families where parents can concentrate on one child. Outdoor activities, in contrast, are activities in which several children of different ages can easily participate.

### Homeschooling

In the present sample of primary school children, all children received materials for homeschooling. The increased amount of children receiving learning materials at a regular basis at t2 compared to t1 and the increased usage of internet platforms, e-mails, and learning videos to provide children with learning materials suggest that support of children by schools has increased during the lockdown. The attitudes towards school were rather positive. Only a very small minority of children was never motivated, never had fun, or was never able to concentrate on schoolwork. Regarding the amount of schoolwork, an average duration of 2.5 hours per day seems long, considering that homeschooling can be a challenging and stressful situation for everyone involved. However, two hours is much less than the time of pedagogical work under normal learning conditions (i.e. at school), indicating that the homeschooling system in its present form cannot replace learning at school. The average duration of schoolwork in the present study was also lower than the duration of daily homeschooling reported by parents of 1- to 5 graders in Czech Republic (2–4 hours/day) [16]. This observation might suggest that either the homeschooling system or the motivation of children and parents might differ between countries or cultures.

We observed a significant decrease in children’s motivation to do schoolwork as the closing of school and, therefore, the necessity of homeschooling continued. This finding indicates that homeschooling is an increasing challenge for primary school pupils and their parents. This assumption is consistent with another recent study in which parents in seven different European countries reported the negative quality of homeschooling and the negative impact of homeschooling on their children [26].

Importantly, the present analyses showed that receiving learning materials on a regular basis is associated with a higher daily duration of schoolwork. Receiving new learning materials on a regular basis may help to make learning objectives (e.g. completion of final learning tasks) more concrete, to better structure learning, and to better estimate the workload.

As expected, we observed that the attitudes towards school were less positive in children from socially weaker families. This finding shows that homeschooling represents a greater challenge for families where parents may have more difficulties in providing adequate support to their children, e.g., due to limited coping strategies, poorer working conditions or less education.

### Limitations

The underrepresentation of participants from lower social strata might limit the generalizability of the findings to the whole population of children. Since the assessments before the lockdown (during the regular visits in the LIFE Child study) and during the lockdown were not identical, we could not investigate any associations or differences between the behavior before and during the lockdown. Regarding homeschooling, we did not assess whether children had the necessary technology equipment at home (computers, printers, Internet) or whether schools enabled interactive learning through videoconferencing. Such differences might influence the motivation and success of homeschooling and explain differences found between children with different SES.

## Conclusions

The loss of childcare and schooling at daycare facilities and schools represents an exceptional situation for children and parents, which, with increasing length, can be associated with a decline in (inter)active indoor behavior and a decreased motivation to do schoolwork. Homeschooling cannot replace education at school and is further impaired by a lack of structuring by the school. For socially weaker families, homeschooling and educational support at home pose a particular challenge.

## Supporting information

S1 Table Questions of the online surveys analyzed in the present project. (PDF)
