# Supplementary material for: Loss of childcare and classroom teaching during the Covid-19-related lockdown in spring 2020: A longitudinal study on consequences on leisure behavior and schoolwork at home
Source: PLoS One. 2021 Mar 2;16(3):e0247949. doi: 10.1371/journal.pone.0247949 (PMC7924794; doi:10.1371/journal.pone.0247949)
Supplement: S1 Table — (PDF) [file pone.0247949.s001.pdf]

Table 1. Questions of the online surveys analyzed in the present project

Article: Loss of childcare and classroom teaching during the Covid-19-related lockdown in spring 2020: A longitudinal study on consequences on leisure behavior and schoolwork at home

Authors: Tanja Poulain, Christof Meigen, Carolin Sobek, Peggy Ober, Ulrike Igel, Antje Körner, Wieland Kiess, Mandy Vogel

German (original)	English (translation)
**Childcare situation before and during lockdown**	
1. Welche Einrichtung besucht Ihr Kind?<br>***Antwortoptionen:*** Krippe[a], Kindergarten[a], Grundschule, weiterführende Schule, Berufsschule, Kind ist kein Schüler mehr	1. What facility does your child attend?<br>***Answer options:*** Nursery (for children younger than three years)[a], Kindergarten[a], Elementary school, Secondary school, Professional school, child is no longer a student
2. Wer übernimmt hauptsächlich die Betreuung durch den Ausfall von Schule/Kindergarten?<br>***Antwortoptionen:*** hauptsächlich Mutter, hauptsächlich Vater, Mutter/Vater zu etwa gleichen Teilen, Großeltern, Notbetreuung, das Kind benötigt keine Betreuung[b], andere[b]	2. Who is mainly responsible for childcare due to the closure of schools and kindergartens?<br>***Answer options:*** mainly mother, mainly father, mother/father equally, grandparents, emergency care, child does not need care[b], other[b]
3. Wie viele Kinder leben während des Lockdowns in Ihrem Haushalt?<br>***Antwortformat:*** offen	3. How many children live in your household during the lockdown?<br>***Response format:*** open
**Leisure behavior**	
1. Wie oft ging Ihr Kind in der letzten Woche folgenden Tätigkeiten nach? Puzzlen, Basteln, Handarbeit, Malen, Zeichnen[c]<br>***Antwortoptionen:*** mindestens 1x/Tag, mindestens 3x/Woche, mindestens 1x/Woche, seltener als 1x/Woche[d], nie[d]	1. How often did your child perform the following activities in the last week? Jigsaw puzzles, handicraft, painting[c]<br>***Response options:*** at least 1x per day, at least 3x per week, at least 1x per week, less than 1x per week[d], never[d]
2. Wie oft ging Ihr Kind in der letzten Woche folgenden Tätigkeiten nach? Gesellschaftsspiele<br>***Antwortoptionen:*** mindestens 1x/Tag, mindestens 3x/Woche, mindestens 1x/Woche, seltener als 1x/Woche[d], nie[d]	2. How often did your child perform the following activities in the last week? Board games<br>***Response options:*** at least 1x per day, at least 3x per week, at least 1x per week, less than 1x per week[d], never[d]
3. Wie oft ging Ihr Kind in der letzten Woche folgenden Tätigkeiten nach? Sport drinnen<br>***Antwortoptionen:*** mindestens 1x/Tag, mindestens 3x/Woche, mindestens 1x/Woche, seltener als 1x/Woche[d], nie[d]	3. How often did your child perform the following activities in the last week? Indoor sports<br>***Response options:*** at least 1x per day, at least 3x per week, at least 1x per week, less than 1x per week[d], never[d]
4. Wie oft ging Ihr Kind in der letzten Woche folgenden Tätigkeiten nach? Spiel draußen<br>***Antwortoptionen:*** mindestens 1x/Tag, mindestens 3x/Woche, mindestens 1x/Woche, seltener als 1x/Woche[d], nie[d]	4. How often did your child perform the following activities in the last week? Playing outside<br>***Response options***: at least 1x per day, at least 3x per week, at least 1x per week, less than 1x per week[d], never[d]
**Media use[e]**	
1. Wie lange hat sich Ihr Kind in der letzten Woche pro Tag durchschnittlich mit folgenden Dingen beschäftigt? Filme, Serien<br>***Antwortoptionen:*** gar nicht, 30 Minuten, 1-2 Stunden, 3-4 Stunden, > 4 Stunden	1. How long did your child spend on average per day during the last week on the following things? Movies, series<br>***Response options:*** not at all, 30 minutes, 1-2 hours, 3-4 hours, > 4 hours
2. Wie lange hat sich Ihr Kind in der letzten Woche pro Tag durchschnittlich mit folgenden Dingen	2. How long did your child spend on average per day during the last week on the following things? Video

beschäftigt? Computerspiele<br>***Antwortoptionen:*** gar nicht, ca. 30 Minuten, ca. 1-2 Stunden, ca. 3-4 Stunden, > 4 Stunden	games<br>***Response options:*** not at all, 30 minutes, 1-2 hours, 3-4 hours, > 4 hours
3. Wie lange hat sich Ihr Kind in der letzten Woche pro Tag durchschnittlich mit folgenden Dingen beschäftigt? Vorlesen lassen/gemeinsam lesen<br>***Antwortoptionen:*** gar nicht, ca. 30 Minuten, ca. 1-2 Stunden, ca. 3-4 Stunden, > 4 Stunden	3. How long did your child spend on average per day during the last week on the following things? Receiving reading/reading together<br>***Response options:*** not at all, 30 minutes, 1-2 hours, 3-4 hours, > 4 hours
4. Wie lange hat sich Ihr Kind in der letzten Woche pro Tag durchschnittlich mit folgenden Dingen beschäftigt? Musik/Radio/Hörspiele hören<br>***Antwortoptionen:*** gar nicht, ca. 30 Minuten, ca. 1-2 Stunden, ca. 3-4 Stunden, > 4 Stunden	4. How long did your child spend on average per day during the last week on the following things? Listening to music/radio/radio plays<br>***Response options:*** not at all, 30 minutes, 1-2 hours, 3-4 hours, > 4 hours
**Support of homeschooling by school**[f]	
1. Bekommt Ihr Kind von seiner Schule Lernmaterial für zu Hause?<br>***Antwortoptionen:*** ja, nein	1. Does your child receive learning materials for home from his/her school?<br>***Response options:*** yes, no
2. In welcher Form erhält Ihr Kind von seiner Schule Lernmaterial?<br>***Antwortoptionen:*** Online-Plattformen, E-Mail, Lernvideos, andere	2. How do teachers provide your child with learning materials?<br>***Response options:*** Online platforms, e-mail, learning videos, other
3. Wie oft erhält Ihr Kind von seiner Schule Lernmaterial?<br>***Antwortoptionen:*** regelmäßig (ca. 1x pro Woche), unregelmäßig (seltener als 1x pro Woche)	3. How often does your child receive learning materials from school?<br>***Response options:*** regularly (at least 1x per week), irregularly (less often than 1x per week)
**Attitudes towards schoolwork**[f]	
1. Wie viele Stunden hat sich Ihr Kind in der letzten Woche im Durchschnitt pro Schultag mit Schulaufgaben beschäftigt?<br>***Antwortformat:*** offen	1. In the last week, how many hours on average did your child spend doing schoolwork per school day?<br>***Response format:*** open
2. Konnte sich Ihr Kind in der letzten Woche gut auf seine Schulaufgaben konzentrieren?<br>***Antwortoptionen:*** nie, selten, manchmal, oft, immer	2. In the last week, was your child able to concentrate well on his or her schoolwork?<br>***Response options:*** never, seldom, quite often, very often, always
3. Hat Ihr Kind die Schulaufgaben in der letzten Woche gern gemacht?<br>***Antwortoptionen:*** nie, selten, manchmal, oft, immer	3. In the last week, did your child enjoy doing schoolwork?<br>***Response options:*** never, seldom, quite often, very often, always
4. Konnte sich Ihr Kind in der letzten Woche gut selbst motivieren, die Schulaufgaben zu erledigen?<br>***Antwortoptionen:*** nie, selten, manchmal, oft, immer	4. In the last week, was your child able to motivate himself/herself well to complete schoolwork?<br>***Response options:*** never, seldom, quite often, very often, always

[a]Response options “nursery (for children younger than three years)” and “kindergarten” were combined to one category “nursery/kindergarten”
[b]Response options “child does not need care” and “other” were combined to “other”
[c]In the manuscripts, these activities are referred to as handicrafts.
[d]Response options less than “1x per week” and “never” were combined to one category “never”.
[e]At t1, separate questions were asked for weekday and weekend. As we observed no differences between media use on weekday and weekend, only media use on weekdays was analyzed. At t2, the questions were not separated for weekday and weekend.
[f]Assessed in the subsample of primary school children only (n = 102).
